# Benzodiazepine high‐doses: The need for an accurate definition

**DOI:** 10.1002/mpr.1888

**Published:** 2021-07-31

**Authors:** Jean‐Marc Cloos, Christopher Y. S. Lim Cow, Valéry Bocquet

**Affiliations:** ^1^ Department of Psychiatry Hôpitaux Robert Schuman Luxembourg Luxembourg; ^2^ Department of Public Health Competence Centre in Methodology and Statistics Luxembourg Institute of Health Luxembourg; ^3^ Unité de Soutien Méthodologique CHU La Réunion Saint‐Denis France

**Keywords:** anxiolytics, benzodiazepine, high‐dose use, hypnotics, long‐term use

## Abstract

**Objectives:**

A clear definition of what we understand of high‐dose misuse or of a ‘markedly increased dose’ (as stated by the DSM‐5) is important and past definitions may be inadequate. The aim of this review is to describe the different definitions used and to test these definitions for their accuracy.

**Methods:**

A narrative PubMed literature review was conducted based on articles published between 1 January 1990 and 31 December 2020 describing benzodiazepines (in MeSH Terms or MeSH Major Topic) and high‐dose (or high‐dosage). Specific definitions were applied to a population sample to show how definitions affect high‐dose benzodiazepine prevalence.

**Results:**

Multiples of an equivalent‐diazepam dose or of the World Health Organization ‘defined daily dosage’ were used more frequently than the overstep of the recommended maximum therapeutic dosage as a cut‐off point.

**Conclusion:**

High‐dose use is rare but the prevalence in the general population varies among studies, mainly due to different definitions, making both clinical and epidemiological comparisons between studies difficult. Defining a high‐dose user as a person who takes at least a higher dose than the maximum usual therapeutic dose over a defined period of time therefore appears to be clinically more consistent.

**Abbreviations**
BDZ: benzodiazepines
DDD: defined daily dose
DSM: diagnostic and statistical manual of mental disorders
EDD: equivalent diazepam dose
HDU: high‐dose user
MDD: main daily dose
MeSH: medical subject headings
PTSD: posttraumatic stress disorder
SDD: standard daily dose
UTD: usual therapeutic dose
WHO: World Health Organization

## 1 INTRODUCTION

DSM‐5 does not any longer distinguish between ‘abuse’ and ‘dependence’ diagnoses. It combines these terms into ‘substance use disorder’ which ranges from mild to severe, based on the number of criteria met. The former DSM‐IV sedative, hypnotic or anxiolytic abuse (305.40) now needs two to three symptoms to be quoted as a ‘mild substance use disorder’ in the DSM‐5, while dependence of these substances (304.10) requires four to five symptoms to be characterized as moderate and over six symptoms if severe. The symptoms that classify a sedative, hypnotic or anxiolytic use disorder according to DSM‐5 are listed in Table 1.

**TABLE 1:** DSM‐5 diagnostic criteria for sedative‐, hypnotic‐ or anxiolytic‐related disorders

A problematic pattern of sedative, hypnotic, or anxiolytic use leading to clinically significant impairment or distress, as manifested by at least two of the following, occurring within a 12‐month period:
1. Sedatives, hypnotics, or anxiolytics are often taken in larger amounts or over a longer period than was intended
2. There is a persistent desire or unsuccessful efforts to cut down or control sedative, hypnotic or anxiolytic use
3. A great deal of time is spent in activities necessary to obtain the sedative, hypnotic or anxiolytic; use the sedative, hypnotic or anxiolytic; or recover from its effects
4. Craving, or a strong desire or urge to use the sedative, hypnotic or anxiolytic
5. Recurrent sedative, hypnotic or anxiolytic use resulting in a failure to fulfil major role obligations at work, school or home (e.g., repeated absences from work or poor work performance related to sedative, hypnotic or anxiolytic use; sedative‐, hypnotic‐ or anxiolytic‐related absences, suspensions, or expulsions from school; neglect of children or household)
6. Continued sedative, hypnotic or anxiolytic use despite having persistent or recurrent social or interpersonal problems caused or exacerbated by the effects of sedatives, hypnotics or anxiolytics (e.g., arguments with a spouse about consequences of intoxication; physical fights)
7. Important social, occupational or recreational activities are given up or reduced because of sedative, hypnotic or anxiolytic use
8. Recurrent sedative, hypnotic or anxiolytic use in situations in which it is physically hazardous (e.g., driving an automobile or operating a machine when impaired by sedative, hypnotic or anxiolytic use)
9. Sedative, hypnotic or anxiolytic use is continued despite knowledge of having a persistent or recurrent physical or psychological problem that is likely to have been caused or exacerbated by the sedative, hypnotic or anxiolytic
10. Tolerance, as defined by either of the following:
a) A need for markedly increased amounts of the sedative, hypnotic or anxiolytic to achieve intoxication or desired effect
b) A markedly diminished effect with continued use of the same amount of the sedative, hypnotic or anxiolytic
**Note**: This criterion is not considered to be met for individuals taking sedatives, hypnotics or anxiolytics under medical supervision.
11. Withdrawal, as manifested by either of the following:
a) The characteristic withdrawal syndrome for sedatives, hypnotics or anxiolytics
b) Sedatives, hypnotics or anxiolytics (or a closely related substance, such as alcohol) are taken to relieve or avoid withdrawal symptoms
**Note**: This criterion is not considered to be met for individuals taking sedatives, hypnotics or anxiolytics under medical supervision.
*Specify* if: In early remission: After full criteria for sedative, hypnotic or anxiolytic use disorder were previously met, none of the criteria for sedative, hypnotic or anxiolytic use disorder have been met for at least 3 months but for less than 12 months (with the exception that Criterion A4, “Craving, or a strong desire or urge to use the sedative, hypnotic or anxiolytic,” may be met) In sustained remission: After full criteria for sedative, hypnotic or anxiolytic use disorder were previously met, none of the criteria for sedative, hypnotic or anxiolytic use disorder have been met at any time during a period of 12 months or longer (with the exception that Criterion A4, “Craving, or a strong desire or urge to use the sedative, hypnotic or anxiolytic,” may be met) *Specify* if: In a controlled environment: This additional specifier is used if the individual is an environment where access to sedatives, hypnotics or anxiolytics is restricted *Specify* current severity: 305.40 (F13.10) Mild: Presence of 2‐3 symptoms304.10 (F13.20) Moderate: Presence of 4‐5 symptoms304.10 (F13.20) Severe: Presence of 6 or more symptoms

*Note:* American Psychiatric Association. Diagnostic and Statistical Manual of Mental Disorders (DSM‐5®). American Psychiatric Pub, 2013.

Among the group of hypnotics, sedatives and other anxiolytics, benzodiazepines are the most commonly used substances, but a clear definition of abuse of and addiction to these drugs is not settled. Due to their wide‐ranging effects, their field of application is very large: they are often used in general medicine, psychiatry, neurology, internal medicine, and in the field of anaesthesia.

For decades, benzodiazepines have been recommended as the standard treatment of anxiety and insomnia (Egan, 1999; Kurko et al., 2015; Laux & Puryear, 1984; Zandstra et al., 2002). Benzodiazepine withdrawal, rebound, overuse and abuse often limit their use in clinical practice (Moher et al., 2009). Several articles showed that the consequences of benzodiazepines use differs according to their type: anxiolytic or hypnotic (Cloos et al., 2015; Silberman et al., 2020), and to the dose a patient uses.

Benzodiazepines are generally well‐tolerated, and their toxic threshold is very far from their therapeutic threshold. However, their use can cause several problems, depending on the dose and duration of the prescription, individual sensitivity, and their different characteristics. There are differences between abuse, misuse, addiction and dependence (physical, psychological and/or social), but they all have clinical significance (Silberman et al., 2020). There is a significant correlation between the dosage and duration of consumption and the risk of developing a physical dependence (Kan et al., 1997). Scales have been developed to measure the severity of dependence (Cuevas et al., 2000).

Benzodiazepine (BDZ) dependence has been traditionally defined either as ‘low‐dose’ or as ‘high‐dose’ (Laux & Puryear, 1984). Withdrawal symptoms, rebound symptoms, and persistent postwithdrawal disorders may however occur in several drug classes after decrease, discontinuation, or switch of psychotropic medications (Cosci & Chouinard, 2020), and some patients develop a tolerance to certain substances and progressively increased dosages. Since the severity of the disorder is now based on the number of symptoms which meet the criteria, neither tolerance nor withdrawal is necessary for a diagnosis of a substance use disorder. Symptoms of tolerance and withdrawal seen during appropriate medical treatment with prescribed BDZs are no longer taken into consideration when diagnosing a sedative, hypnotic or anxiolytic substance use disorder and patients complying with the prescribed dosages should not be diagnosed as ‘addicted’, if withdrawal or tolerance occurs as a result of the medical treatment. The concept of low‐dose ‘iatrogenic’ dependence may therefore be considered as obsolete, even if these patients may require a progressive reduction of the dosages to avoid withdrawal symptoms.

Tolerance is now defined in DSM‐5 by one of the following symptoms: a ‘markedly increased dose’ of the substance to achieve the desired effect or a ‘markedly reduced effect’ when the usual dose is taken. It is necessary to agree on what we understand by ‘markedly increased dose’ and at what point the dosage taken by the patient is considered to be inadequate. The term ‘high‐dose’ has been commonly used by many authors, but definitions vary widely in the literature, and there appears to be no clear consensus (Egan, 1999; Kurko et al., 2015; Zandstra et al., 2002) thus limiting comparability between studies. As the use of high BDZ doses is often the result of the development of a tolerance, a clinical cut‐off point on when the prescription becomes improper is needed.

Clinically, there may be different reasons for long‐term use of BDZs and the progressive switch to higher dosages: decrease or loss of clinical effects, tolerance (pharmacokinetic, i.e., higher dosages are required to keep the same results; pharmacodynamic, i.e., refractory to dose increase), resistance to treatment, craving and addiction, the type of disorder (e.g., panic disorder generally requires higher doses). Liebrenz, Schneider et al. (2015) identified the following reasons to explain continued and high dose BDZ use: (1) to cope with symptoms of psychological distress or mental disorder other than substance use, (2) to manage symptoms of physical or psychological discomfort associated with somatic disorder, (3) to alleviate symptoms of substance‐related disorders, and (4) for recreational purposes, that is, sensation‐seeking and other social reasons. The studies selected however frequently examine the average use over a certain period of time, and it remains unclear if the high dose was taken from the very beginning or later in time.

Psychiatric and somatic comorbidity in patients with severe BDZ dependence is frequent. These patients very often have other psychoactive substance use disorders (e.g., opioids, cocaine or alcohol dependence), suffer from insomnia, anxiety disorders (especially panic disorder and generalized anxiety disorder) and affective disorders. BDZ abuse and dependence is also prevalent in several personality disorders (antisocial, avoidant, borderline, obsessive‐compulsive, histrionic and dependent personality) as well as in rheumatological, neurological and cardiovascular disorders (Busto et al., 1996; Couvée et al., 2002; Martínez‐Cano et al., 1999).

The aim of this article is to dress a list of the different definitions of increased BDZ use found in the literature, to identify prevalence, and to test these definitions for their accuracy in clinical practice, in order to provide a better understanding of what should be considered a BDZ ‘high‐dose’ or a ‘markedly increased dose’.

## 2 METHODS

### 2.1 Search strategy

We followed a standard protocol for this review according to the Preferred Reporting Items of Systematic reviews and Meta‐Analyses guidelines (Moher et al., 2009). We conducted a narrative PubMed literature review selecting articles published in English, German and French between 1 January 1990 and 31 December 2020 that described high‐dose consumption of BDZs in humans. The choice of these languages and the study period was due to the need of being able to gather and understand the articles’ full text, and not solely rely on the English abstract, rarely sufficient to define the relevance of the article.

The BDZs studied consisted of two subgroups: (a) anxiolytic BDZs (N05BA) and (b) hypnotic BDZs (N05CD). We included clonazepam as an antiepileptic (N03AE01), but excluded the BDZ derivatives (‘Z‐Drugs’, N05CF): zolpidem, zaleplon, and zopiclone are non‐BDZs, since they lack the benzene and diazepine rings, even if their abuse and addiction potential, especially for zolpidem, is very similar to BDZs (Tamburin et al., 2021).

The search design was developed with a medical librarian. We constructed a PubMed Boolean search string with the following keywords: ((((‘benzodiazepines’[MeSH Terms]) AND ((high‐dose) OR high‐dosage))) OR ((((benzodiazepines/administration and dosage[MeSH Major Topic]))) AND ((high‐dose) OR high‐dosage))) OR ((‘benzodiazepines’[MeSH Major Topic]) AND ((high‐dose) OR high‐dosage)). In addition, we realized a manual search in the reference lists of the selected papers that did not retrieve any other includable article.

### 2.2 Eligibility criteria

The eligibility of studies was estimated using standardized inclusion and exclusion criteria. We included papers in the dates of publication and languages described earlier. In a first step, we read the titles and the abstracts to select the papers to be included in our review. Only studies on chronic high‐dose usage associated with BDZs in humans were selected. Articles concerning intravenous (IV) midazolam used only in anaesthesia were also filtered out. In case of doubt on the inclusion of a specific reference in the final selection, in a second step, the decision to select or reject the article for our review was made by reading its full text.

### 2.3 Data extraction and synthesis

We classified the articles according to the definitions of high‐dose BDZ used and discussed the different cut‐off points found in the literature. Selected definitions were applied to population data from our published study (Cloos et al., 2015) to show their effects on high‐dose use prevalence rates.

## 3 RESULTS

**FIGURE 1:**
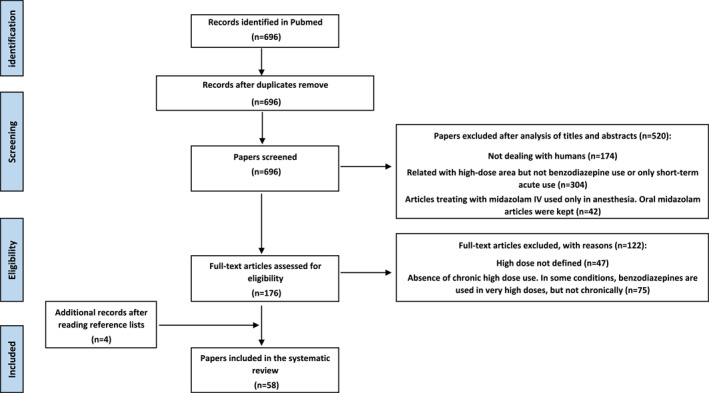
PRISMA flowchart of search strategy and abstract screening

A total of 696 articles were found using our PubMed Boolean search string. 520 articles were excluded by reading the abstract and the title. Of the total, 122 further articles were excluded after full‐text reading, leaving a total of 54 articles (Figure 1). Four more articles listed in the references of the retained selection did not meet our Boolean search criteria, but have been included since they were of particular additional interest.

The BDZ high‐dose definitions proposed in the selected articles were then classified according to their methodology. Characteristics of the 58 selected articles are described in Table 2. Amongst these articles, 2 were from 1985 to 1990, 6 from 1990 to 1995, 3 from 1995 to 2000, 6 from 2000 to 2005, 4 from 2005 to 2010, 8 from 2010 to 2015, and 29 from 2015 onwards. Of these 58 articles, 32 came from Europe, 7 from the United States, 6 from Asia (Taiwan, Japan, South Korea), 4 from Switzerland, 3 from Canada, 2 from the United Kingdom, 1 from South America, 1 from Australia, and 2 are not applicable. These papers came mainly from epidemiological studies (*n* = 13) and selected patients (*n* = 20). Length of follow‐up is mainly at least one year (*n* = 30) or from a 3‐month to 1‐year duration (*n* = 16). High‐dose definitions usually come from studies comprising at least 50 people (*n* = 43), largely adults (*n* = 43) in outpatient treatment (*n* = 28) using several BDZ (*n* = 53). Table 2 also highlights the studies examining the subgroups of benzodiazepines (anxiolytics and hypnotics) separately, and not as a whole. Not all benzodiazepines are the same and undifferentiated studies may provide insufficient data of potential risk of a specific BDZ (Cosci et al., 2015; Zito, 2015).

**TABLE 2:** Description of the characteristics of the included studies

Characteristics of the included studies (number of studies)	References
Country of the study	
Asia and Australia (*n* = 7)	Harnod et al., 2015; Hata et al., 2018; Kim et al., 2017; Rintoul et al., 2013; Takeshima et al., 2016; Tien et al., 2020; Wen et al., 2014
Canada (*n* = 3)	Egan et al., 2001; Sketris et al., 1985; Sullivan & Sellers, 1992
France (*n* = 2)	Etchepare et al., 2016; Imbert et al., 2016
Germany (*n* = 4)	Brinkers et al., 2016; Holzbach et al., 2010; Janhsen et al., 2015; Kaendler et al., 1996
BeNeLux (*n* = 2)	Cloos et al., 2015; Voshaar et al., 2003
Mediterranean Europe (*n* = 12)	Faccini et al., 2016, 2019; Federico et al., 2020, 2017; Lekka et al., 1997, 2002; Lugoboni et al., 2020, 2014, 2018; Martinez‐Cano et al., 1996; Quaglio et al., 2012; Tamburin et al., 2017
Scandinavia (*n* = 12)	Andenæs et al., 2016; Bajwah et al., 2018; Bjerrum et al., 1994; Fredheim et al., 2019, 2020; Fride Tvete et al., 2015; Johansson et al., 2003; Neutel et al., 2012; Nordfjaern et al., 2014, 2013; Sidorchuk et al., 2018; Vorma et al., 2003
South America (*n* = 1)	Moreno‐Gutíerrez et al., 2020
Switzerland (*n* = 4)	Liebrenz, Gehring et al., 2015; Liebrenz, Schneider et al., 2015; Liebrenz et al., 2016a, 2016b
UK (*n* = 2)	Perera & Jenner, 1987; Seivewright & Dougal, 1993
USA (*n* = 7)	Conry et al., 2009; Ellinwood et al., 1990; Hanlon et al., 2009; Hermos et al., 2007, 2005; Kroll et al., 2016; Soumerai et al., 2003
Not applicable (*n* = 2)	Alexander & Perry, 1991; Teboul & Chouinard, 1991
Type of study	
Systematic review (*n* = 5)	Alexander & Perry, 1991; Brinkers et al., 2016; Janhsen et al., 2015; Kim et al., 2017; Teboul & Chouinard, 1991
Randomized controlled trial (*n* = 2)	Conry et al., 2009; Ellinwood et al., 1990
Epidemiological study (*n* = 13)	Andenæs et al., 2016; Bajwah et al., 2018; Egan et al., 2001; Etchepare et al., 2016; Federico et al., 2017; Hanlon et al., 2009; Harnod et al., 2015; Holzbach et al., 2010; Johansson et al., 2003; Nordfjaern et al., 2014, 2013; Rintoul et al., 2013; Soumerai et al., 2003
National or large registry (*n* = 9)	Cloos et al., 2015; Fredheim et al., 2019, 2020; Fride Tvete et al., 2015; Moreno‐Gutíerrez et al., 2020; Neutel et al., 2012; Sidorchuk et al., 2018; Takeshima et al., 2016; Wen et al., 2014
Local register (*n* = 8)	Bjerrum et al., 1994; Faccini et al., 2016; Federico et al., 2020; Hata et al., 2018; Hermos et al., 2007; Lugoboni et al., 2020, 2014; Tamburin et al., 2017;
Selected patients (*n* = 20)	Faccini et al., 2019; Hermos et al., 2005; Imbert et al., 2016; Kaendler et al., 1996; Kroll et al., 2016; Lekka et al., 1997, 2002; Liebrenz, Gehring et al., 2015; Liebrenz, Schneider et al., 2015; Liebrenz et al., 2016a, 2016b; Lugoboni et al., 2018; Martinez‐Cano et al., 1996; Perera & Jenner, 1987; Quaglio et al., 2012; Seivewright & Dougal, 1993; Sullivan & Sellers, 1992; Tien et al., 2020; Vorma et al., 2003; Voshaar et al., 2003
Selected physicians (*n* = 1)	Sketris et al., 1985
Length of follow‐up	
Less than 3 months (*n* = 4)	Andenæs et al., 2016; Bjerrum et al., 1994; Ellinwood et al., 1990; Etchepare et al., 2016
3 months to 1 year (*n* = 16)	Conry et al., 2009; Egan et al., 2001; Faccini et al., 2016; Federico et al., 2017; Holzbach et al., 2010; Johansson et al., 2003; Quaglio et al., 2012; Seivewright & Dougal, 1993; Lekka et al., 1997; Lugoboni et al., 2014, Neutel et al., 2012; Perera & Jenner, 1987; Sketris et al., 1985; Tamburin et al., 2017; Vorma et al., 2003; Wen et al., 2014
More than 1 year (*n* = 30)	Bajwah et al., 2018; Cloos et al., 2015; Faccini et al., 2019; Fredheim et al., 2019, 2020; Fride Tvete et al., 2015; Hanlon et al., 2009; Harnod et al., 2015; Hata et al., 2018; Hermos et al., 2007, 2005; Imbert et al., 2016; Janhsen et al., 2015; Kaendler et al., 1996; Kroll et al., 2016; Lekka et al., 2002; Liebrenz, Gehring et al., 2015; Liebrenz, Schneider et al., 2015; Liebrenz et al., 2016a, 2016b; Moreno‐Gutíerrez et al., 2020; Nordfjaern et al., 2014, 2013; Rintoul et al., 2013; Sidorchuk et al., 2018; Soumerai et al., 2003; Sullivan & Sellers, 1992; Takeshima et al., 2016; Tien et al., 2020; Voshaar et al., 2003
Not applicable (*n* = 8)	Alexander & Perry, 1991; Brinkers et al., 2016; Federico et al., 2020; Kim et al., 2017; Lugoboni et al., 2020, 2018; Martinez‐Cano et al., 1996; Teboul & Chouinard, 1991

Characteristics of study population	References
By age group	
Young people (*n* = 3)	Conry et al., 2009; Fredheim et al., 2020; Sidorchuk et al., 2018
Adults (*n* = 43)	Andenæs et al., 2016; Bajwah et al., 2018; Bjerrum et al., 1994; Brinkers et al., 2016; Cloos et al., 2015; Ellinwood et al., 1990; Faccini et al., 2016, 2019; Federico et al., 2020, 2017; Fredheim et al., 2019; Fride Tvete et al., 2015; Harnod et al., 2015; Hata et al., 2018; Hermos et al., 2007, 2005; Holzbach et al., 2010; Imbert et al., 2016; Johansson et al., 2003; Kroll et al., 2016; Lekka et al., 1997, 2002; Liebrenz, Gehring et al., 2015; Liebrenz, Schneider et al., 2015; Liebrenz et al., 2016a, 2016b; Lugoboni et al., 2020, 2014, 2018; Nordfjaern et al., 2014, 2013; Martinez‐Cano et al., 1996; Perera & Jenner, 1987; Quaglio et al., 2012; Rintoul et al., 2013; Seivewright & Dougal, 1993; Soumerai et al., 2003; Sullivan & Sellers, 1992; Takeshima et al., 2016; Tamburin et al., 2017; Vorma et al., 2003; Voshaar et al., 2003; Wen et al., 2014
Elderly (*n* = 5)	Egan et al., 2001; Etchepare et al., 2016; Hanlon et al., 2009; Neutel et al., 2012; Tien et al., 2020
All age group (*n* = 1)	Moreno‐Gutíerrez et al., 2020
Not available (*n* = 1)	Kaendler et al., 1996
Not applicable (*n* = 5)	Alexander & Perry, 1991; Janhsen et al., 2015; Kim et al., 2017; Sketris et al., 1985; Teboul & Chouinard, 1991
Size of the study population	
<50 (*n* = 9)	Federico et al., 2017; Kaendler et al., 1996; Liebrenz, Gehring et al., 2015; Liebrenz, Schneider et al., 2015; Liebrenz et al., 2016a, 2016b; Quaglio et al., 2012; Seivewright & Dougal, 1993; Sullivan & Sellers, 1992
≥50 (*n* = 43)	Andenæs et al., 2016; Bajwah et al., 2018; Bjerrum et al., 1994; Cloos et al., 2015; Conry et al., 2009; Egan et al., 2001; Ellinwood et al., 1990; Etchepare et al., 2016; Faccini et al., 2016, 2019; Federico et al., 2020, 2017; Fredheim et al., 2019, 2020; Fride Tvete et al., 2015; Hanlon et al., 2009; Harnod et al., 2015; Hata et al., 2018; Hermos et al., 2007, 2005; Holzbach et al., 2010; Imbert et al., 2016; Johansson et al., 2003; Kroll et al., 2016; Lekka et al., 2002, 1997; Lugoboni et al., 2020, 2014, 2018; Martinez‐Cano et al., 1996; Moreno‐Gutíerrez et al., 2020; Neutel et al., 2012; Nordfjaern et al., 2014, 2013; Perera & Jenner, 1987; Rintoul et al., 2013; Sidorchuk et al., 2018; Soumerai et al., 2003; Tamburin et al., 2017; Takeshima et al., 2016; Tien et al., 2020; Voshaar et al., 2003; Wen et al., 2014
Not applicable (*n* = 6)	Alexander & Perry, 1991; Brinkers et al., 2016; Janhsen et al., 2015; Kim et al., 2017; Sketris et al., 1985; Teboul & Chouinard, 1991
Type of patients	
Inpatient (*n* = 14)	Bajwah et al., 2018; Etchepare et al., 2016; Faccini et al., 2016, 2019; Federico et al., 2020, 2017; Johansson et al., 2003; Hata et al., 2018; Lugoboni et al., 2020, 2014, 2018; Quaglio et al., 2012; Sullivan & Sellers, 1992; Tamburin et al., 2017
Outpatient (*n* = 28)	Andenæs et al., 2016; Bjerrum et al., 1994; Cloos et al., 2015; Conry et al., 2009; Egan et al., 2001; Ellinwood et al., 1990; Fride Tvete et al., 2015; Hanlon et al., 2009; Harnod et al., 2015; Hermos et al., 2007, 2005; Holzbach et al., 2010; Imbert et al., 2016; Kaendler et al., 1996; Kroll et al., 2016; Lekka et al., 1997, 2002; Martinez‐Cano et al., 1996; Moreno‐Gutíerrez et al., 2020; Neutel et al., 2012; Nordfjaern et al., 2014, 2013; Perera & Jenner, 1987; Rintoul et al., 2013; Soumerai et al., 2003; Takeshima et al., 2016; Vorma et al., 2003; Voshaar et al., 2003
In‐ and out‐patient (*n* = 10)	Fredheim et al., 2019, 2020; Liebrenz, Gehring et al., 2015; Liebrenz, Schneider et al., 2015; Liebrenz et al., 2016a, 2016b; Seivewright & Dougal, 1993; Sidorchuk et al., 2018; Tien et al., 2020; Wen et al., 2014
Not applicable (*n* = 6)	Alexander & Perry, 1991; Brinkers et al., 2016; Janhsen et al., 2015; Kim et al., 2017; Sketris et al., 1985; Teboul & Chouinard, 1991
Category of benzodiazepines	
Hypnotics (*n* = 8)	Andenæs et al., 2016; Cloos et al., 2015; Ellinwood et al., 1990; Faccini et al., 2019; Martinez‐Cano et al., 1996; Sullivan & Sellers, 1992; Takeshima et al., 2016; Wen et al., 2014
Anxiolytics (*n* = 6)	Cloos et al., 2015; Hermos et al., 2007; Imbert et al., 2016; Martinez‐Cano et al., 1996; Rintoul et al., 2013; Takeshima et al., 2016
Not applicable (*n* = 47)	Alexander & Perry, 1991; Bajwah et al., 2018; Bjerrum et al., 1994; Brinkers et al., 2016; Conry et al., 2009; Egan et al., 2001; Etchepare et al., 2016; Faccini et al., 2016; Fang et al., 2009; Federico et al., 2020, 2017; Fredheim et al., 2020; Fride Tvete et al., 2015; Hanlon et al., 2009; Harnod et al., 2015; Hata et al., 2018; Hermos et al., 2005; Janhsen et al., 2015; Johansson et al., 2003; Kaendler et al., 1996; Kim et al., 2017; Kroll et al., 2016; Lekka et al., 1997, 2002; Liebrenz, Gehring et al., 2015; Liebrenz, Schneider et al., 2015; Liebrenz et al., 2016a, 2016b; Lugoboni et al., 2020, 2014, 2018; Moreno‐Gutíerrez et al., 2020; Neutel et al., 2012; Nordfjaern et al., 2014, 2013; Perera & Jenner, 1987; Quaglio et al., 2012; Seivewright & Dougal, 1993; Sidorchuk et al., 2018; Sketris et al., 1985; Soumerai et al., 2003; Tamburin et al., 2017; Teboul & Chouinard, 1991; Tien et al., 2020; Vorma et al., 2003; Voshaar et al., 2003
BDZ studied	
One (*n* = 5)	Conry et al., 2009; Faccini et al., 2019; Imbert et al., 2016; Rintoul et al., 2013; Sullivan & Sellers, 1992
Several (*n* = 53)	Alexander & Perry, 1991; Andenæs et al., 2016; Bajwah et al., 2018; Bjerrum et al., 1994; Brinkers et al., 2016; Cloos et al., 2015; Egan et al., 2001; Ellinwood et al., 1990; Etchepare et al., 2016; Faccini et al., 2016; Federico et al., 2020, 2017; Fredheim et al., 2019, 2020; Fride Tvete et al., 2015; Hanlon et al., 2009; Harnod et al., 2015; Hata et al., 2018; Hermos et al., 2007, 2005; Holzbach et al., 2010; Janhsen et al., 2015; Johansson et al., 2003; Kaendler et al., 1996; Kim et al., 2017; Kroll et al., 2016; Lekka et al., 1997, 2002; Liebrenz, Gehring et al., 2015; Liebrenz, Schneider et al., 2015; Liebrenz et al., 2016a, 2016b; Lugoboni et al., 2020, 2014, 2018; Martinez‐Cano et al., 1996; Moreno‐Gutíerrez et al., 2020; Neutel et al., 2012; Nordfjaern et al., 2014, 2013; Perera & Jenner, 1987; Quaglio et al., 2012; Seivewright & Dougal, 1993; Sidorchuk et al., 2018; Sketris et al., 1985; Soumerai et al., 2003; Takeshima et al., 2016; Tamburin et al., 2017; Teboul & Chouinard, 1991; Tien et al., 2020; Vorma et al., 2003; Voshaar et al., 2003; Wen et al., 2014

## 4 DISCUSSION

### 4.1 Benzodiazepine high‐dose definitions used in research

The following major definitions were identified by examining the research articles:

- A specific equivalent dose of diazepam.
- A specific daily, weekly or yearly World Health Organization (WHO) defined daily dose (DDD) fraction.
- A percentile of an average daily dose.
- A combination of length and dose used.
- Summated standard daily dose.
- A maximum therapeutic dose recommended by the manufacturer.

Some articles mentioned high‐dose BDZ use but did not define it (Lader, 2011; Liebrenz et al., 2010). Specific definitions were also applied for special populations (e.g., the elderly) or individual substances. A summary of the different definitions is given in Table 3.

**TABLE 3:** Different definitions given for high‐dose BDZ use and their references

Definition for high‐dose use (number of studies)	Complete definition	Rationale	Duration	Specific population	Patients' consequences
≥5 mg diazepam (*n* = 1) (Hata et al., 2018)	/	/	/	/	/
≥10 mg diazepam (*n* = 1) (Voshaar et al., 2003)	/	From statistical analysis: high dosage use = best independent predictor of relapse by dichotomizing daily dosage at a 10 mg EDD.	/	/	/
≥20 mg diazepam (*n* = 3)	“High‐dose consumption” or “high‐dose dependence” without the use duration being defined (Janhsen et al., 2015).	/	Mean prescribed daily dose (PDD) averages were calculated every three months for analysis (Moreno‐Gutíerrez et al., 2020).	“High‐dose benzodiazepine addiction for ≥60 years”: daily use of more than 20 mg of a EDD, without the notion of use duration (Brinkers et al., 2016).	Janhsen et al. (2015) recommend hospital admission when starting withdrawal treatment
≥30 mg diazepam (*n* = 4)	High‐dose BDZ prescription: a daily EDD of ≥30 mg (Kroll et al., 2016, citing an older article from Cushman & Benzer, 1980).High‐dose users: taking BDZs in non‐therapeutic doses as an over 30 mg EDD (Quaglio et al., 2012).	The EDD appears to have been calculated using these median daily doses and are not taken from the manufacturers’ recommendations.	Quaglio et al. (2012): 6 months.	The EDDs were added together when patients were using multiple BDZ agents (Kroll et al., 2016). However, the potency equivalence between BDZ agents is not clearly established.	Quaglio et al. (2012) considered taking BDZs in non‐therapeutic doses as an over 30 mg EDD. High‐dose users meeting this criterion were admitted to a stationary detoxification programme if >6‐month‐consumption and impossibility to reduce dosage.
≥40 mg diazepam (*n* = 8)	“High‐dose withdrawal” when including patients who have been ingesting doses of BDZ greater than the EDD of 40 mg/day for a period of more than 8 months (Alexander & Perry, 1991) Liebrenz et al. (2015–2016) definition: ● took BDZs for an extended period of time (without defining “long‐term”) ● for a higher than 40 mg EDD, and/or ● those who had an otherwise problematic use of BDZs (such as substance mixing, repeated dose escalation, recreational use, euphoric effect enhancement, illegal acquisition strategies or experiences of negative social consequences). Users with dosage taken by these patients had been at least a 40 mg EDD prior to sudden cessation (Seivewright & Dougal, 1993). “Very high dosage”: at least 100 EDDs (Soumerai et al., 2003).	No reason for Alexander and Perry (1991)From an expert panel, including two academic psychopharmacologists (Soumerai et al., 2003).	Alexander and Perry (1991): ≥8 months. Liebrenz et al. (2015–2016): extended period of time (without defining “long‐term”). Seivewright and Dougal (1993): no information. Vorma et al. (2003): long‐term without any definition of long. Soumerai et al. (2003): minimum of 2 years as long‐term (continuing) recipients.	Soumerai et al. (2003) defined high daily dosage as at least 20 EDDs for patients ≥65 years and at least 40 EDDs for younger patients.	/
≥50 mg diazepam (*n* = 8)	“High‐dose dependency”: intake of more than 50 EDDs in a clinical withdrawal study (Kaendler et al., 1996). In Italy, the maximum approved dose of diazepam for extra‐hospital use is 10 mg/day. A research team from Verona considers an intake exceeding at least five times the maximum daily recommended dose as “high‐dose” (Lugoboni et al., 2014), that is, 50‐mg diazepam. “High‐dose abuse”: ● >50 mg diazepam/day intake over an extended period of time (>6 months), (Faccini et al., 2019; Federico et al., 2020; Lugoboni et al., 2020, 2018) and/or ● an otherwise problematic use of BDZs, such as combining BDZs, escalating dosage, using BDZs for recreational purposes, or obtaining BDZs illegally (Faccini et al., 2016; Federico et al., 2017; Tamburin et al., 2017).	/	Lugoboni et al. (2014): >6 months.	/	/
≥0.5 DDD (*n* = 4)	“High‐dose BDZ user”: a person taking 0.5 DDD/day during a 1‐year period (Nordfjaern et al., 2014, 2013) based on the recommendation that BDZs should not usually be used beyond 2–4 weeks (National Institute for Clinical Excellence & Britain, 2004), that an intake over 180 days should be qualified as “long‐term” (Zandstra et al., 2002) and that “180 prescription days or above within one year” had been previously considered “long‐term BDZ use” (Fang et al., 2009). “Frequent” BDZ user: a person taking over 180 DDD over a 1‐year period (Andenæs et al., 2016)	From statistical analysis: Cox regression analysis (Takeshima et al., 2016).	/	/	/
≥1 DDD (*n* = 2)	The prescribed daily dose (PDD)/DDD ratio was used to assess whether appropriate doses were used. High dose was defined as a PDD/DDD ratio >1 (Tien et al., 2020).	/	/	Elderly population in Canada: prescription defined as a high daily dose if the prescribed dosage is higher than 1 DDD for that particular BDZ (Egan et al., 2001).	/
≥1.5 DDD (*n* = 2)	Sidorchuk et al. (2018) distinguish between “regular users” (≥0.5 to <1.5 DDD/day for >1 year) and “heavy users” (≥1.5 DDD/day for >1 year).	From statistical analysis: quartile methods (Harnod et al., 2015). 1.5 DDD corresponds to the highest category of DDD through the selection of four groups.	/	/	/
≥2 DDD (*n* = 2)	● “Excessive users” (Fride Tvete et al., 2015). ● “Major benzodiazepine consumers” (Bjerrum et al., 1994).	/	Over 3 months within a 5‐year period (Fride Tvete et al., 2015). Dose is mentioned but no duration by Bjerrum et al. (1994).	/	/
≥3 DDD (*n* = 1)	“Long‐term high‐dose user.”	/	Wen et al. (2014): 90 days.	/	/
≥4 DDD (*n* = 1)	“High‐dose psychotropic drug dependence”.	/	Johansson et al. (2003): no length.	/	/
A fixed dosage of DDD over a certain period of time	Over 100 DDD per year (Fredheim et al., 2019, 2020).	/	/	/	/
A percentile of an average daily dose (*n* = 2)	High‐dose BDZ users (Hermos et al., 2007).	Based on quartiles: ● Comparison of 5%‐ highest average daily doses to 25% and 75% percentiles (Hermos et al., 2007). ● HDU as the average daily dose for the longest treatment episode in the top 10th percentile for each agent (Hermos et al., 2007).	/	/	/
A combination of length and dose used (*n* = 1) (Holzbach et al., 2010)	Dependent: ● Orange code attributed if the equivalent daily dose of diazepam was between 5 and 10 mg of diazepam. ● Red code if between 10 and 15 mg. ● Black code if over 15 mg.	/	Prescriptions longer than 6 months.	/	/
Summated standard daily dose (SDD) (*n* = 1) (Hanlon et al., 2009)	For every user, multiplication of the number of dosage forms by medication strength, then dividing it by the minimum effective dose per day recommended. The result corresponds to a summated standard daily dose (SDD).	/	/	The studied population being older adults, the authors relied on a geriatric pharmacotherapy reference for establishing the minimum effective daily dose (Semla et al., 2016). Low dose treatment was then defined as under one SDD and high‐dose as over three SDD.	/
A maximum therapeutic dose recommended by the manufacturer (*n* = 5)	BDZ over‐prescription: users receiving a higher dose than the manufacturers' recommended maximum daily dosage over a certain period of time (Sketris et al., 1985).Use of the British National Formulary to identify high‐dose users taking the maximum or doses higher than recommended for more than 6 months (Perera & Jenner, 1987). For those taking several BDZs, the fractions of the dose of each BDZ taken were divided by the maximum recommended dose for each one of the BDZ or using the maximum dose recommendations of the Greek National Pharmaceutical Organization (Brayfield, 2017; Lekka et al., 2002) and the quotients were added together to give an index. Same methodology with Cloos et al. (2015) where consumed dose was compared to the yearly maximum usual therapeutic dosages (taken from the Martindale and Micromedex Drugdex®). In cases of multiple BDZs, yearly doses were divided by the yearly equivalent maximum usual therapeutic dose of each BDZ.	/	/	/	/
Selection length (6 months, 1 year) and references used for the highest recommended dosages (*n* = 1)	Use of several sources to define high and therapeutic dose: clinical experience, manufacturer's recommendations and data in the literature (Martinez‐Cano et al., 1996).	/	/	/	/
50% dose reduction in the elderly (*n* = 5)	“Inappropriate BDZ use”: dosage of over 9 DDDs per week or a total of more than 300 DDDs over the year for anxiolytic BDZs in elderly (Neutel et al., 2012). “High‐dosage”: at least a diazepam equivalent dose of 20 mg per day for elderly patients (≥65 years) and at least 40 mg per day for patients under 65 years (Soumerai et al., 2003).	/	/	Most of the studies, however, do not consider the recommended dose reduction in the elderly when defining high‐dose use, thus underestimating abuse in the elderly.	/
Specific high dose definitions (*n* = 6)	Clobazam high‐dose treatment in children is defined as over 10 mg by Conry et al. (2009). “High‐doses” in adults: 30 mg of flurazepam (Ellinwood et al., 1990), 15 mg of oxazepam (Bajwah et al., 2018), 20 mg of oxazepam (Imbert et al., 2016), 2 mg of alprazolam (Rintoul et al., 2013), and 5 mg of triazolam (Sullivan & Sellers, 1992). Kim et al. (2017) define for example high‐dose temazepam treatment as over 1640 mg/year of this molecule (equal to over 82 DDDs/year).	/	/	/	/

#### 4.1.1 A specific equivalent dose of diazepam

Converting BDZs into an equivalent dose of diazepam (EDD) is common in clinical practice and useful in BDZ withdrawal. There is often a range rather than a unique value for a specific BDZ, for example, 10 mg of diazepam is equivalent to 20–50 mg of tetrazepam, yet there is no standard conversion table, and diazepam dose equivalence tables can only be found in some of the articles using the EDD method (e.g., Alexander & Perry, 1991; Brinkers et al., 2016; Kaendler et al., 1996).

As seen by these diverse diazepam cut‐off dose levels, the EDD methodology is not sufficiently accurate for prevalence studies, but is frequently used in clinical settings. Liebrenz et al. states that there is no universally accepted definition of high‐dose BDZ dependence (Liebrenz, Gehring, et al., 2015; Liebrenz, Schneider, et al., 2015; Liebrenz et al., 2016a, 2016b). Once converted in diazepam equivalent, authors use different values to define high‐dosage, from a greater than one EDD of 10 mg (Voshaar et al., 2003) up to 50 mg (Faccini et al., 2016, 2019; Federico et al., 2017, 2020; Kaendler et al., 1996; Lugoboni et al., 2020, 2014, 2018; Tamburin et al., 2017). In previous studies, patients with an EDD of 20–500 mg were hospitalized for withdrawal (Harrison et al., 1984; Janhsen et al., 2015).

#### 4.1.2 A specific daily, weekly or yearly DDD fraction

According to the WHO, the DDD is a unit developed to measure ‘the average maintenance dose per day for a drug used for its main indication in adults’ (WHO, 2003). It does not necessarily correspond to the recommended or prescribed daily dose and is often a compromise between different doses in various countries. There may be insufficient clinical support and the equivalent dose of 10 mg of diazepam may represent 6.25%–200% of the DDD, depending on the specific BDZ. The DDD definitions may be arbitrary (e.g., due to the prescription habits in a specific country) and they are sometimes reconsidered, thus varying in time. The DDD for clonazepam, for example, may be inappropriate (Islam et al., 2014), since DDD of 8 mg is listed for that drug, where the usual clinical dose is 0.5 mg. To define high‐dose use, the authors rely on a specific daily, weekly or yearly DDD fraction in their studies, as described in Table 3.

To reflect the mean BDZ dose of all the different BDZs by one parameter, Kan et al. (1997) divided the mean daily BDZ dose (MDD) by the defined daily BDZ dose (DDD). While the basic definition idea is similar (an index over one), prevalence may differ according to selection length (6 months, 1 year) and the references used for the highest recommended dosages. Fredheim et al. (2019, 2020) define high dose use of benzodiazepines and benzodiazepine‐related hypnotics, in an adolescent population, as receiving more than 100 DDDs in 1 year.

Specific attention should be given to the ageing population (≥65 years) in which a 50% reduction of the recommended adult dose is generally recommended (Etchepare et al., 2016; Nakra & Grossberg, 1986; Teboul & Chouinard, 1991) or a dose selection at the low end of the adult dosage range (Hanlon et al., 2009). In certain clinical conditions, higher doses than the maximum recommended daily dose may be adequate. There is also no clear consensus on what length of high‐dose use should be considered inappropriate.

#### 4.1.3 Example

An example of how prevalence varies with different high‐dose definitions is given in Table 4. We compared the two definitions most commonly used for HDU, that is, an equivalent dose of diazepam (EDD10, cut‐off point: 20 mg/day, i.e., >7300 mg/year) and a yearly DDD fraction (cut‐off point: 2 DDDs/day, i.e., >730 DDDs/year) with the definition of surpassing the usual therapeutic dose (>365 UTD/year). The data was drawn from BDZ prescription claims from a general population sample of the Grand‐Duchy of Luxembourg from 1995 to 2006 (*N* = 247,170). We observed higher estimation of HDU when applying EDD10 or DDD definitions, compared to UTD. The reason is that the EDD10 and DDD definitions include patients as ‘high‐dose users’ that are actually still using BDZs within therapeutic limits. Cut‐off points of 20 mg (two DDDs) and 30 mg (three DDDs) are prone to an overestimation of the HDU prevalence. Two DDDs often stay within therapeutic limits, and even three DDDs are still under the highest recommended prescription margin for some BDZs (e.g., for alprazolam, clorazepate and diazepam).

**TABLE 4:** Proportion of high‐dose users in a benzodiazepine prescribed population (*N* = 247,170)

Age (years)	High‐dose use	High‐dose use definition	1996	2001	2006
<65	EDD10	2 EDD10/day over 1 year	5.2%	5.1%	4.8%
	DDD	2 DDD/day over 1 year	4.8%	4.7%	4.4%
	UTD	>UTDmax/day over 1 year	4.6%	3.8%	3.4%
≥65	EDD10	1 EDD10/day over 1 year	19.6%	20.8%	22.2%
	DDD	1 DDD/day over 1 year	18.3%	19.6%	20.8%
	UTD	>50% UTDmax/day over 1 year	15.2%	14.8%	15.6%

Abbreviations: DDD, defined daily dose; EDD10, approximative equivalent dose of 10 mg of diazepam (1 EDD10 = 10 mg of diazepam); UTDmax, usual maximum therapeutic dose.

On the other hand, a 50% dose restriction is usually recommended in the elderly. If applied to the studies' definitions, 15%–20% of BDZ users aged 65 and over would be identified as high‐dose users, while the proportion of those aged under 65 (with a higher cut‐off point) is close to 5%. If we consider the complete insured population, the prevalence of high‐dose use is around 0.8%.

What method should be used for determining long‐term high‐dose BDZ exposure? Diazepam equivalent doses (EDD), WHO DDD or the manufacturer's maximum therapeutic doses? As shown in our example, the EDD and the DDD methods tend to overestimate prevalence. Criterion 1 of the new DSM‐5 definition of the sedative‐, hypnotic‐ or anxiolytic‐related disorder is met when the substance is ‘often taken in larger amounts or over a longer period than was intended’. Tolerance is defined as ‘needing a markedly increased dose to achieve intoxication or desired effect, or by markedly diminished effect with continued use of the same amount’. However, ‘symptoms of tolerance and withdrawal occurring during appropriate medical treatment with prescribed medications are specifically not counted when diagnosing a substance use disorder. The appearance of normal, expected pharmacological tolerance and withdrawal during the course of medical treatment has been known to lead to an erroneous diagnosis of “addiction”, even when these were the only symptoms present. Individuals whose only symptoms are those that occur as a result of medical treatment (i.e., tolerance and withdrawal as part of medical care when the medications are taken as prescribed) should not receive a diagnosis solely on the basis of these symptoms’. If the ‘larger amounts’ fall into therapeutic limits and a specific time frame during an adequate medical treatment, should we then diagnose a pathological high‐dose use? Where do we most usefully draw the line between ‘usual therapeutic’ exposure and ‘markedly increased’ (or excessive) exposure? What are ‘larger amounts’? What is a ‘longer period than intended’?

Sometimes specific psychiatric or neurological disorders require, for a short period of time (days or 1–2 weeks), a dosage substantially higher than the maximum recommended therapeutic dosage (see Table 5), thus creating a bias when the study period is set too short (e.g., 1 or 3 months) and these short‐term very‐high‐dose users risk being included in an abuse group. A 1‐year period to study long‐term or high‐dose will reduce such biases (Kurko et al., 2015; Zandstra et al., 2002).

**TABLE 5:** Characteristics of 24 benzodiazepines

Drug	EDD10	DDD	UTD		HTD
			Min	Max	
Anxiolytics					
Alprazolam	0.5	1.00	0.25	4.00	10.00
Bromazepam	6	10.00	3.00	18.00	60.00
Clobazam	20	20.00	10.00	30.00	80.00
Clonazepam	0.5–2	8.00	0.50	8.00	20.00
Clorazepate	15–20	20.00	15.00	60.00	90.00
Clotiazepam	5–10	15.00	5.00	15.00	60.00
Cloxazolam	1–2	9.00	1.00	4.00	12.00
Diazepam	10	10.00	4.00	40.00	Variable
Ketazolam	15–30	30.00	15.00	60.00	135.00
Loflazepate	1–2	2.00	1.00	3.00	4.00
Lorazepam	1–2	2.50	1.00	6.00	10.00
Nordazepam	10–20	15.00	5.00	15.00	20.00
Oxazepam	20	50.00	15.00	120.00	300.00
Prazepam	10–20	30.00	10.00	60.00	60.00
Tetrazepam	20–50	100.00	25.00	150.00	400.00
Hypnotics					
Brotizolam	0.25–0.5	0.25	0.13	0.25	0.50
Flunitrazepam	0.5–1	1.00	0.50	1.00	2.00
Flurazepam	15–30	30.00	15.00	30.00	30.00
Loprazolam	1–2	1.00	0.50	1.00	2.00
Lormetazepam	1–2	1.00	0.50	2.00	4.00
Midazolam (IV)	5–7.5	20.00	2.00	20.00	0.35^a^
Nitrazepam	5–10	5.00	2.50	10.00	20.00
Temazepam	20	20.00	10.00	20.00	40.00
Triazolam	0.25–0.5	0.25	0.13	0.25	0.50

Abbreviations: DDD, daily defined dose (World Health Organization); EDD10, approximative equivalent dose of 10 mg of diazepam; HTD, highest therapeutic dosage, exceptionally recommended (in severe cases); UTD, usual therapeutic dosage (minimal et maximal doses); IV, intravenous.

^a^ Expressed in mg per body weight in kg.

Several chronic psychiatric disorders may also require a prolonged use of BDZs, if not treatable by other pharmacologic or nonpharmacological means, for example, general anxiety disorder, panic disorder, chronic insomnia, some psychotic or demential states, bipolar affective disorder (clonazepam), epilepsy (clonazepam, nitrazepam) and even BDZ addiction itself, if remaining symptomatic after several earnest but unsuccessful attempts of withdrawal (Collège des Médecins du Québec, 1997/1998). Prescribed BDZ doses for the treatment of these chronic disorders are likely to remain within therapeutic limits, but some of them already fall in the high‐dose‐users category with the EDD or the DDD methods.

A ‘higher than the usual therapeutic dosage’ therefore appears to be a better way to define a ‘markedly increased dosage’ and a usage longer than a year should become the standard for studying ‘long‐term use’. Problematic high‐dose use may then be defined as an ‘over 1‐year usage of a markedly increased dose (i.e., higher than the usual therapeutic dosage)’.

### 4.2 The need of an international consensus

There is no international consensus on the maximum usual therapeutic doses for all substances. For example, the maximum dose recommended by the National Pharmaceutical Organization in Greece (used as cut‐off points by Lekka et al., 2002) does not, at least for some substances, correspond to maximum dosages proposed by other sources (see Table 2). Standardization of the maximum therapeutic dosages is therefore also necessary when evaluating high‐dose use by the UTD definition. In such a ‘consensus dosage table’, the cut‐off‐point is not necessarily the maximum annual dosage proposed by the manufacturers, but may be set lower by a panel of experts if they consider a longer high‐dose use of the specific BDZ as being harmful. Indeed, the dose level considered by a manufacturer as being safe for 4 weeks (e.g., in the treatment of an anxiety disorder) may eventually become harmful if the drug is taken at that dose for a longer period of time. Thus, one might also argue that a 365‐UTDs‐per‐year cut‐off could be as problematic as maximum levels of diazepam (e.g., 7300 mg per year) or DDDs. In the absence of an international 1‐year‐maximum‐dosage‐consensus, the UTD‐cut‐off‐line could therefore be drawn at a lower level, for example, an annual intake of 100 times the manufacturers' maximum therapeutic dosages.

### 4.3 The need of research on specific benzodiazepines

Instead of grouping all benzodiazepines together, as is done in most of the studies, there is a need to focus on each medication on its own in future research. Hypnotic benzodiazepines show a notably higher risk for high dose usage compared to anxiolytics, as shown by our own research (Cloos et al., 2015) and pointed out by other studies (Martínez‐Cano et al., 1996; Takeshima et al., 2016; Wen et al., 2014).

### 4.4 Strengths and limitations

Our study included only publications from PubMed. Other databases such as EMBASE, SCOPUS and PsycINFO and grey literature (theses and dissertations, research and committee reports, government reports, conference papers, and current research) were not included. We believe that an article research of the PubMed database is sufficiently extensive to illustrate the various definitions of BDZ high‐dose that can be found in the scientific literature. Moreover, this research is the first narrative review of its kind to identify all the used definitions associated with benzodiazepine high‐dose.

## 5 CONCLUSION

There is a lack of an internationally recognized standard in the definition of a high daily BDZ dose, and the DSM‐5 concept of a ‘markedly increased dosage’ remains vague. The DSM‐5 diagnosis of a sedative‐, hypnotic‐ or anxiolytic‐related disorder needs several symptoms of compulsive, drug‐seeking behaviour. Also, prescription medications can be used inappropriately, even in therapeutic dosages. However, studies relying solely on prescription or delivery data lack these additional clinical data allowing a more precise diagnosis.

In the past, researchers considered either a certain equivalent dosage of diazepam or an overrun of the WHO DDD as cut‐off‐points between ‘normal’ and ‘high‐dose’ use. None of these methods is entirely satisfying, and prevalence rates of high‐dose use may be overrated when using these definitions. Especially the 20‐mg‐diazepam‐ and the 2‐DDDs‐limit overestimate high‐dose‐use prevalence by including patients who actually respect dosages within therapeutic limits. These patients should not be considered inappropriate users, and they should not receive a diagnosis of a DSM‐5 sedative‐, hypnotic‐ or anxiolytic‐related disorder. The nonrespect of the maximum therapeutic dosage threshold over a period of 1 year appears to be clinically more adequate when defining BDZ high‐dose abuse. We therefore propose to avoid the terminology of ‘high‐dose use’ in future research. Even the DSM‐5 ‘markedly increased dosage’ is unsatisfying. Referring to an ‘above‐therapeutic‐limit’ use would allow better comparisons between studies. Defining a problematic high‐dose user as a person who received at least a higher dose than the usual yearly maximum therapeutic dose is appropriate when observing a population over a longer period of time and studying continuous users.

Furthermore, BDZs vary a lot as to potential dependence. Hypnotic BDZs show a higher risk for high dose dependence than anxiolytic BDZs, and the odds also vary largely inside the two subgroups. For example, triazolobenzodiazepines, such as alprazolam and triazolam, may be particularly susceptible to long‐term and high‐dose use problems. The clinical differences among BDZs may be based on their chemical structure, their potency, the onset and length of action and/or their active metabolite. Based on these pharmacological aspects, future pharmacoepidemiological studies should therefore assess these substances separately, or at least differentiate between the two subgroups.

## CONFLICT OF INTEREST

The authors declare that they have no competing interests. This study did not need any approval from the Human Studies Committee because it was a review of published data in the public domain. Data was confidentially maintained by previous anonymization of the patient data.

## AUTHOR CONTRIBUTIONS

Valéry Bocquet, Christopher Y. S. Lim Cow and Jean‐Marc Cloos meet the criteria for authorship listed hereafter. They have made substantial contributions to conception and design, or acquisition of data, or analysis and interpretation of data. They have been involved in drafting the manuscript and in revising it critically for important intellectual content. They have given final approval of the version to be published. Each author has participated sufficiently in the work to take public responsibility for appropriate portions of the content. They have agreed to be accountable for all aspects of the work in ensuring that questions related to the accuracy or integrity of any part of the work are appropriately investigated and resolved.

## Data Availability

The data that support the findings of this study are available from the corresponding author upon reasonable request.
